# Ex vivo thyroid fine needle aspirations as an alternative for MALDI-MSI proteomic investigation: intra-patient comparison

**DOI:** 10.1007/s00216-020-03088-4

**Published:** 2020-12-05

**Authors:** Isabella Piga, Giulia Capitoli, Francesca Clerici, Allia Mahajneh, Virginia Brambilla, Andrew Smith, Davide Leni, Vincenzo L’Imperio, Stefania Galimberti, Fabio Pagni, Fulvio Magni

**Affiliations:** 1 Proteomics and Metabolomics Unit, School of Medicine and Surgery, University of Milano - Bicocca, 20854 Vedano al Lambro, Italy; 2 Bicocca Bioinformatics Biostatistics and Bioimaging B4 Center, School of Medicine and Surgery, University of Milano - Bicocca, 20900 Monza, Italy; 3 Pathology, School of Medicine and Surgery, San Gerardo Hospital, ASST, University of Milano - Bicocca, 20900 Monza, Italy; 4 Radiology, San Gerardo Hospital, ASST, 20900 Monza, Italy

**Keywords:** *Real*/ex vivo fine needle aspiration, MALDI-MSI, Thyroid, Similarity score, Proteomics

## Abstract

Fine needle aspiration (FNA) is the reference standard for the diagnosis of thyroid nodules. Matrix-assisted laser desorption/ionization mass spectrometry imaging (MALDI-MSI) has been successfully used to discriminate the proteomic profiles of benign and malignant thyroid FNAs within the scope of providing support to pathologists for the classification of morphologically borderline cases. However, *real* FNAs provide a limited amount of material due to sample collection restrictions. Ex vivo FNAs could represent a valuable alternative, increasing sample size and the power of statistical conclusions. In this study, we compared the *real* and ex vivo MALDI-MSI proteomic profiles, extracted from thyrocyte containing regions of interest, of 13 patients in order to verify their similarity. Statistical analysis demonstrated the mass spectra similarity of the proteomic profiles by performing intra-patient comparison, using statistical similarity systems. In conclusion, these results show that post-surgical FNAs represent a possible alternative source of material for MALDI-MSI proteomic investigations in instances where pre-surgical samples are unavailable or the number of cells is scarce.

**Supplementary Information:**

The online version contains supplementary material available at 10.1007/s00216-020-03088-4.

## Introduction

Fine needle aspiration (FNA) is a widely used procedure for the collection of pre-surgical (*real*) specimens used for the diagnosis of benign and malignant lesions in the pre-surgical setting [1].

In recent years, matrix-assisted laser desorption/ionization (MALDI) mass spectrometry imaging (MSI) has been successfully applied for the molecular characterization of thyroid nodules using both ex vivo and *real* FNAs, demonstrating the striking advantage of employing both sample types [2–4]. Specimens collected during outpatient FNAs should be preferentially used for the routine cytopathological diagnosis, whereas ex vivo FNAs, directly collected from the surgical specimens without causing any inconvenience for the patients, could represent a valuable alternative, for basic research interest and for statistical purposes, to allow a large number of cells to be easily collected, to increase sample size and the power of statistical conclusions. However, when the conventional air-dried smear was used for sample preparation of thyroid FNA for MALDI-MSI proteomic analysis, haemoglobin interference affected the mass spectra quality of both ex vivo and in vivo samples, with an increasing rate of unusable in vivo mass spectra due to sample contamination from neck vasculature during the collection process [5]. Later, this technical issue was overcome using a liquid-based preparation and, as a consequence, the mass spectra of *real* and ex vivo FNA became of comparable quality [5, 6]. DeHoog et al. have shown that these two different collection approaches do not influence the lipid profiles of clusters of thyrocyte cells when collected from the same patient, notwithstanding that the overall signal was observed to be generally higher in ex vivo FNAs [3]. Ciregia et al. compared the bidimensional electrophoresis and enzyme-linked immunosorbent assay (ELISA) results from pre- and post-surgical thyroid FNAs, demonstrating the similar levels of some crucial proteins in the two specimens [7]. The authors concluded that potential biomarker candidates could then be investigated in the post-surgical specimens and the results transferred in the pre-surgical samples [7]. However, the similarity of the overall proteomic profiles of these FNA thyroid samples, from the same patients, has never been fully investigated. Accordingly, in the present study, we compared the intra-patient proteomic profiles of thyroid FNA, evaluating the mass spectra similarity both qualitatively and quantitatively.

## Materials and methods

### Specimen collection and preparation

The study was carried out in accordance with the relevant guidelines and regulations. It was approved by the ASST Monza Ethical Board (Associazione Italiana Ricerca sul Cancro-AIRC-MFAG 2016 Id. 18445, HSG Ethical Board Committee approval October 2016, 27102016) and study participants signed an informed consent.

For the present study, 13 patients with malignant thyroid nodules (9 females and 4 males, with an average age of 53 ± 21 years, and an average nodule size of 3 ± 2 cm), who underwent both ultrasound (US)-guided FNAs and total thyroidectomy at San Gerardo Hospital, were enrolled. Both *real* FNAs and ex vivo post-surgical specimens were collected from each patient. FNAs were performed using a 25-gauge needle and one or two passes per nodule were executed: needle washing from every pass was sent for proteomics MALDI-MSI analysis, whereas ex vivo post-surgical FNAs were collected within 30 min after surgery.

Cytological samples were collected into ThinPrep® CytoLyt solution (Hologic, Marlborough, MA, USA), prepared as previously described [6], and finally transferred as a cytospin spot onto ITO-conductive slides. Samples were stored at − 80 °C until the day of analysis. Before MALDI-MSI analysis, cytological specimens were equilibrated to room temperature, dried under vacuum for 30 min, and a uniform coating of the MALDI matrix sinapinic acid (10 mg/ml in 60:40 acetonitrile:water w/0.2% trifluoroacetic acid) was applied with an optimized method (heated bed at 37 °C, 10 spray cycles set with a matrix density on tissue of 5 μl/cm^2^), using the iMatrixSpray (Tardo GmbH, Subingen, Switzerland) automated spraying system.

### MALDI-MSI analysis

MALDI-TOF-MSI analysis was performed using an ultrafleXtreme MALDI-TOF/TOF (Bruker Daltonik GmbH) in positive ion linear mode, within the *m/z* 3000–20,000 range and using 300 laser shots per spot, with a laser focus setting of 3 medium (diameter of 50 μm) and a raster width of 50 × 50 μm. Protein Calibration Standard I (Bruker Daltonics) that contains a mixture of standard proteins within the mass range of 5730 to 16,950 Da was used for external calibration (mass accuracy ±  30 ppm). Data acquisition and visualization were performed using the Bruker software packages (flexControl 3.4, flexImaging 5.0). Following MALDI-MSI analysis, the MALDI matrix was removed with 70% EtOH, the slides stained with haematoxylin and eosin (H&E), digitally scanned using a ScanScope CS digital scanner (Aperio, Park Center Dr., Vista, CA, USA), and images were co-registered to the MSI datasets in flexImaging for the integration of proteomic and morphological data. Regions of interest (ROIs) containing clusters of thyrocytes without interfering elements were comprehensively annotated by the pathologist (see Supplementary Information (ESM) Fig. S1). Subsequently, virtual microdissection of the MSI datasets was performed using FlexImaging.

### Statistical analysis

Average mass spectra from each ROI of the MALDI-TOF-MSI datasets were exported in CSV format and loaded in the open-source R software v.3.6.0 to perform the pre-processing operations that were carried out using the MALDIquant R package [19]. The spectra were processed by performing baseline subtraction (median method), smoothing (moving average method, half window width 2.5), normalization (total ion current, TIC), peak alignment, and peak picking (*S*/*N* ≥ 6). The open-source software mMass v.5.5 (http://www.mmass.org) was used to confirm mass spectra alignment [20, 21].

Hierarchical clustering analysis (HCA) was used for exploratory purposes. HCA was carried out using the complete linkage method to identify similar clusters on principal components. These components were extracted from the principal component analysis (PCA) as the ones that explained the maximum variance of the original independent variables. These unsupervised analyses were performed using the prcomp and hclust function in the R software.

The mass spectra similarity between ROIs of the same patient were evaluated by using the S4cosine score system that consists of 4 components, i.e. fit, retrofit, cosine, and overlap [6]. The cosine index is defined as the cosine angle between the direction in space of two sequences of intensity signals [22]. Assume two spectra $X={\left({x}_i\right)}_{i=1,\dots, {N}_X}$ and $Y={\left({y}_i\right)}_{i=1,\dots, {N}_Y}$, each *i*^*th*^ peak corresponded a mass-to-charge (*m*/*z*) value, while *N*_*X*_ and *N*_*Y*_ are the total number of *m*/*z* values in *X* and *Y*spectra. The Cosine index is defined as $\frac{X\circ Y}{\left\Vert X\right\Vert \cdotp \left\Vert Y\right\Vert }$, ${\boldsymbol{S}}_C\frac{X\circ Y}{\left\Vert X\right\Vert \cdotp \left\Vert Y\right\Vert }$, where $X\circ Y={\sum}_{i=1}^N{x}_i{y}_i$, $\left\Vert X\right\Vert =\sqrt{\sum_{i=1}^N{x}_i^2}$ and *N* was the total number of *m*/*z* values that were to be used in the comparison. The Cosine index is always non-negative if *X* and *Y* have non-negative intensities and varies between 0 (when the spectra are completely different) and 1 (when the spectra are identical). For the evaluation of fit and retrofit indices, only common peaks with an absolute intensity greater than, or equal to, 0.0003 were retained, whereas for the cosine and the overlap measures, all the peaks detected with a S/N higher than, or equal to, 6 were considered.

The similarity of the *real* and ex vivo ROIs was quantified through the S_4cosine_ comparing the two mass spectra obtained from the same patient (inter-sample comparison). To identify the equivalence interval of the proteomic profiles from the two sources, the range of all the *real* vs. *real* and ex vivo vs. ex vivo ROI comparisons in the same sample (intra-sample comparisons) was used as a gold standard, given that no other recognized reference exists. Quartiles, range, mean, standard deviation (sd), and coefficient of variation (cv) were calculated for the description of the composite score.

## Results

In this study, we investigated *real* (*n* = 13) and ex vivo (*n* = 13) thyroid samples collected from the same patient for a total of 26 samples. Overall, the median number of annotated ROIs in the *real* FNA was 8 (I–III quartiles = 5–9), and the corresponding number was 8 (I–III quartiles = 6–10) in the ex vivo counterparts. The analysis focusing on spectra similarity was performed carrying out inter-sample comparisons and by comparing different ROIs from the same FNA. As such, we performed a median number of 54 inter-sample comparisons (I–III quartiles = 39–64), 28 intra-sample comparisons for *real* FNAs (I–III quartiles = 13–46), and 28 for ex vivo samples (I–III quartiles = 15–45).

### Real and ex vivo biopsy similarity: qualitative evaluation

**Fig. 1:**
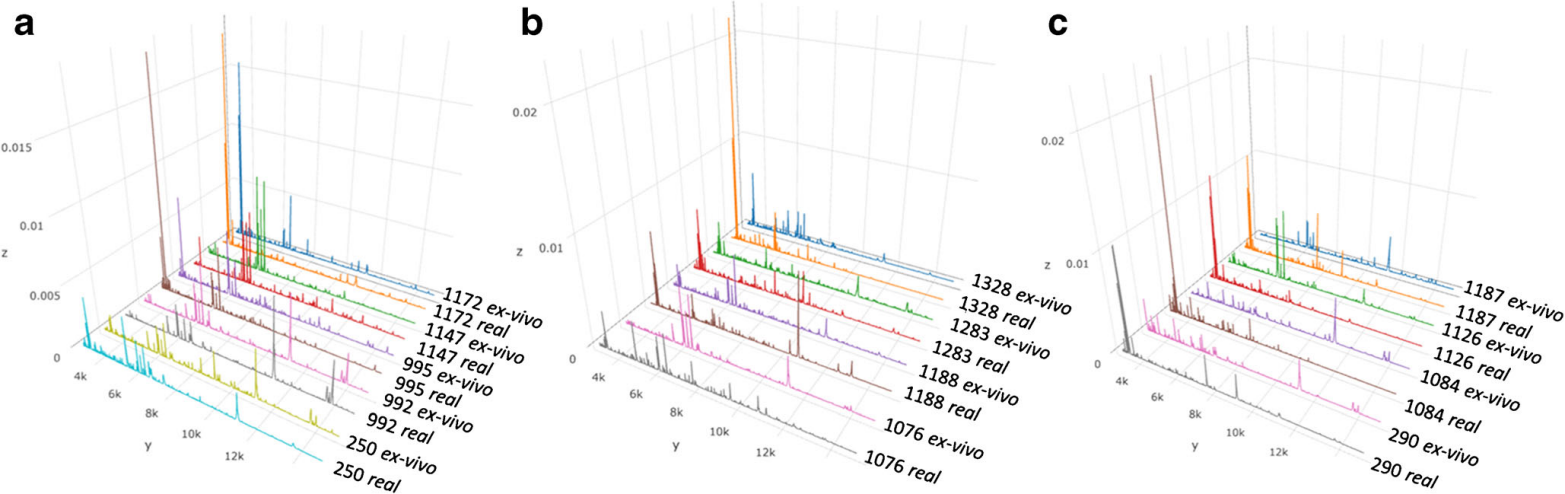
Comparison of the average mass spectra obtained from *real* and ex vivo ROIs from the same patient **a** P250, P992, P995, P1147, P1172; **b** P1076, P1188, P1283, P1328; **c** P290, P1084; P1126, P1187

The average mass spectra of all the *real* and ex vivo ROIs, for each patient, are depicted in Fig. 1, where the profiles are aggregated in three different panels according to their mass spectra similarity, ranging from the highest (Fig. 1a) to the lowest (Fig. 1c). In ESM Fig. S2, a 1-by-1 figure comparing the average mass spectra obtained from the most similar (P1147) and the most dissimilar (P1187) *real* vs. ex vivo spectra is reported to better assess similarity.

**Fig. 2:**
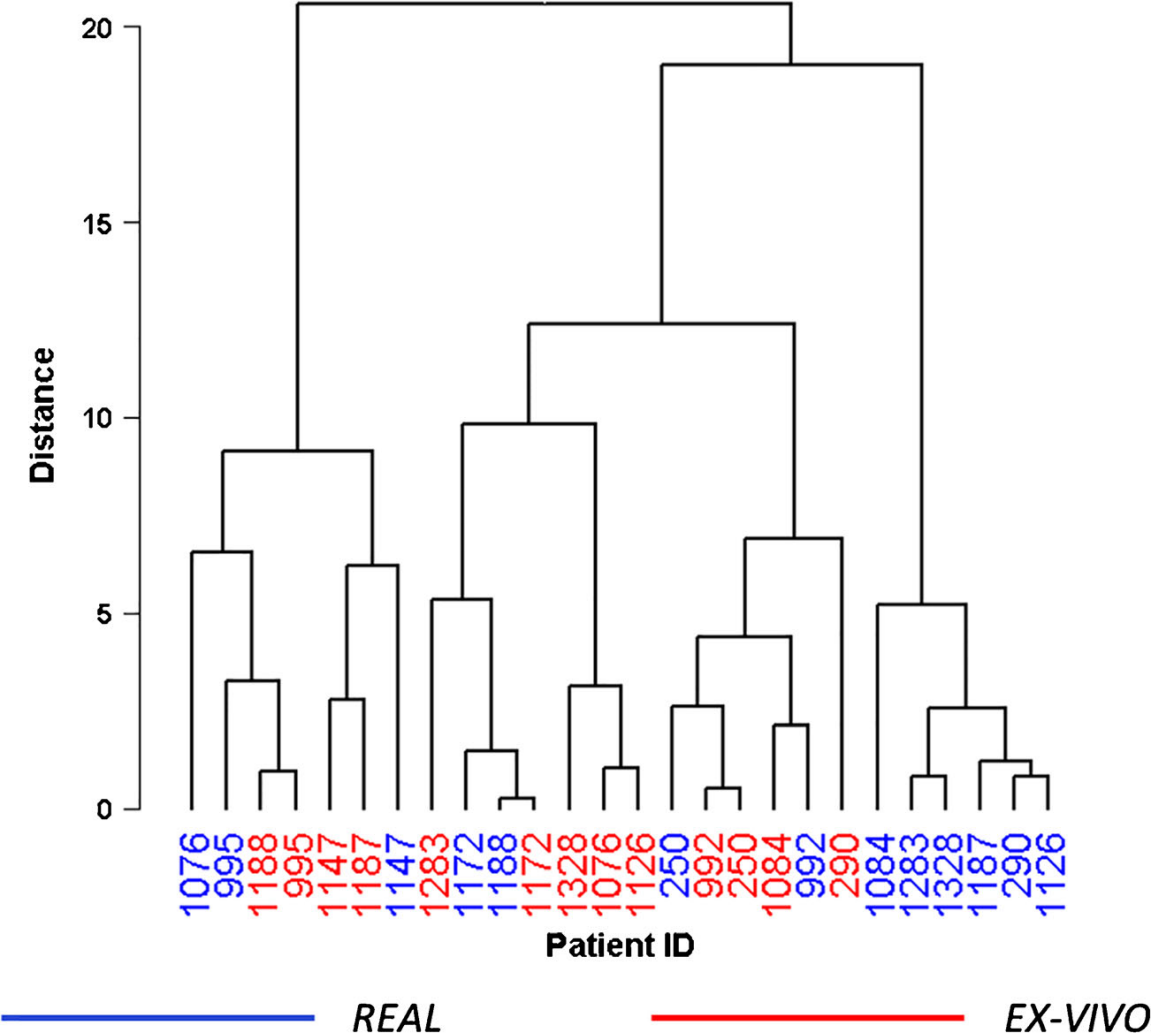
Hierarchical clustering analysis of the average mass spectra obtained from all the *real* (blue) and ex vivo (red) ROIs of each patient

The unsupervised learning method, HCA, was employed to explore mass spectra similarity among the mean spectrum profiles of all the ROIs obtained from *real* and ex vivo samples. HCA revealed no separation between the two classes, highlighting the similarity of the mass spectra (Fig. 2).

In particular, and likewise to what was reported in Fig. 1a, the *real* and ex vivo mass spectra of patients P250, P992, P995, P1147, and P1172, with the highest similarity, are clustered under the same branches of the dendrogram, respectively (Fig. 2). On the other hand, the mean spectrum profiles of *real* biopsies related to P290, P1084, P1126, P1187, P1283, and P1328 are in a separate branch and grouped together in a distinct subgroup (Fig. 2), showing a lower similarity with respect to their ex vivo counterparts. Despite the *real* and ex vivo mass spectra of patients P1076 and P1188 are not clustered together (Fig. 2), the mean spectrum profiles of their *real* biopsies showed a partial overlap with their ex vivo counterparts (Fig. 1b). Moreover, their *real* mass spectra are clustered together with all the ex vivo samples (Fig. 2), showing that they provide comparable proteomics information.

To better understand the observed dissimilarities between *real* and ex vivo samples in some of the patients, we investigated the abundance of the cellular material in the specimens. The 5 samples (P250, P992, P995, P1147, P1172) with the smallest distance in the HCA (Fig. 2), and with the most similar proteomic profiles (Fig. 1a), were also the ones with a similar amount of thyrocyte clusters in the two independent specimens. On the contrary, samples from patients with lower similarity between the proteomic profiles had a lower amount of thyrocyte cells in *real* FNAs.

**Fig. 3:**
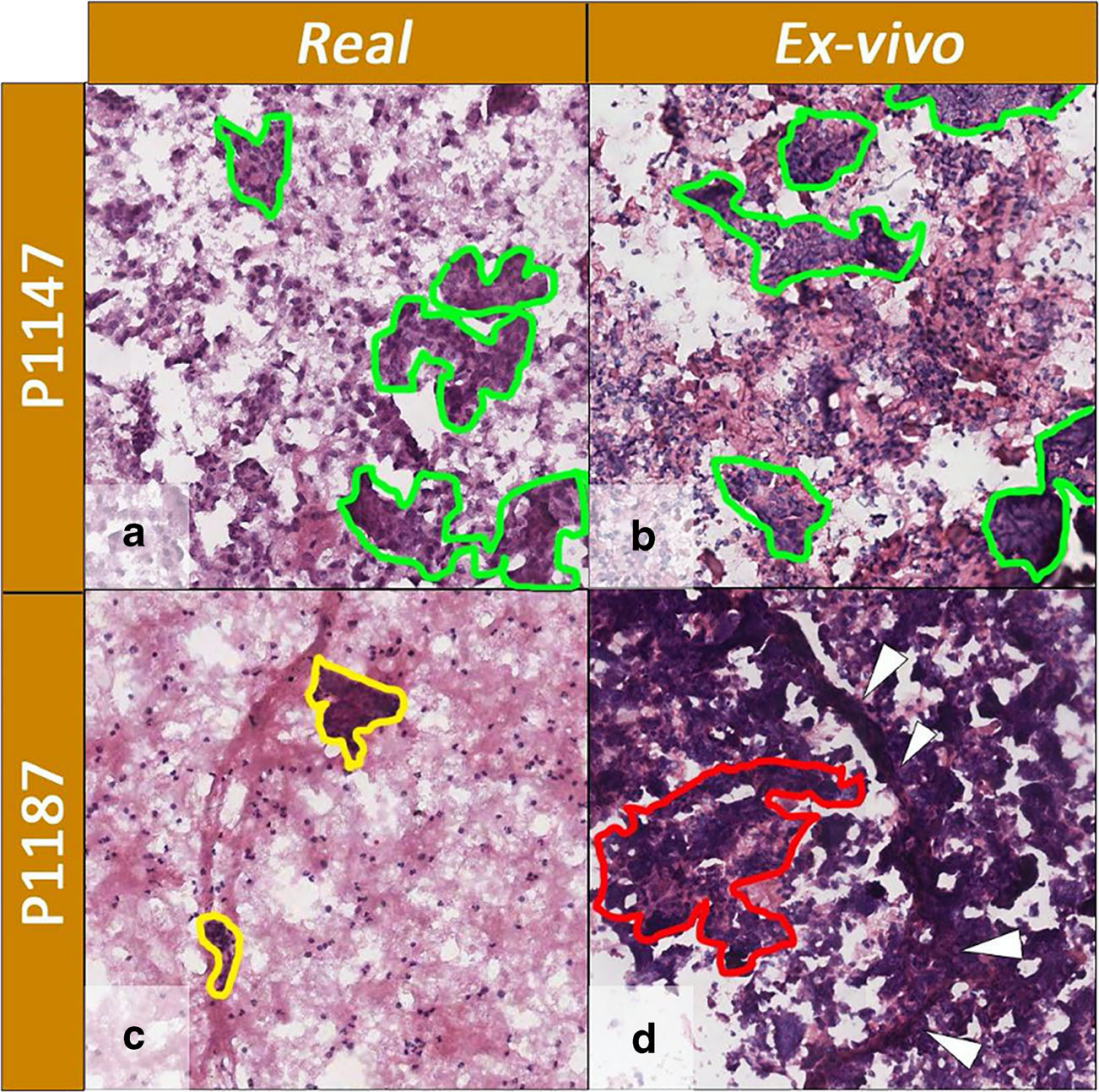
H&E-stained images of *real* (**a**, **c**) and ex vivo (**b**, **d**) samples of patients P1147 and P1187. The comparison between the real (**a**) and ex vivo (**b**) samples obtained from the case P1147 demonstrates a similar cellularity with moderate density of thyrocytes clusters in a similar background (some of the more relevant and similar clusters are highlighted by green lines). On the contrary, the real (**c**) sample from case P1187 differed from its ex vivo (**d**) counterpart for the paucity of analysable clusters (yellow lines) in an almost acellular background as opposite to the highly cellular ex vivo specimen, characterized by numerous vascular cores (white arrowheads) in a rich thyrocyte background (exemplificative cluster in red line). All pictures have a magnification of × 100

Specimens of P1147 have a comparable amount of thyrocyte clusters, whereas P1187 showed paucity of thyrocyte cells and small clusters in the *first* sample (Fig. 3c) compared to the ex vivo counterpart (Fig. 3d). HCA showed that the ex vivo mass spectra of P1147 and P1187 were clustered together with the *real* P1147, reflecting the similar morphology, whereas the *real* P1187 was clustered apart (as previously shown in Fig. 2).

### Real and ex vivo biopsy similarity: quantitative evaluation

**Fig. 4:**
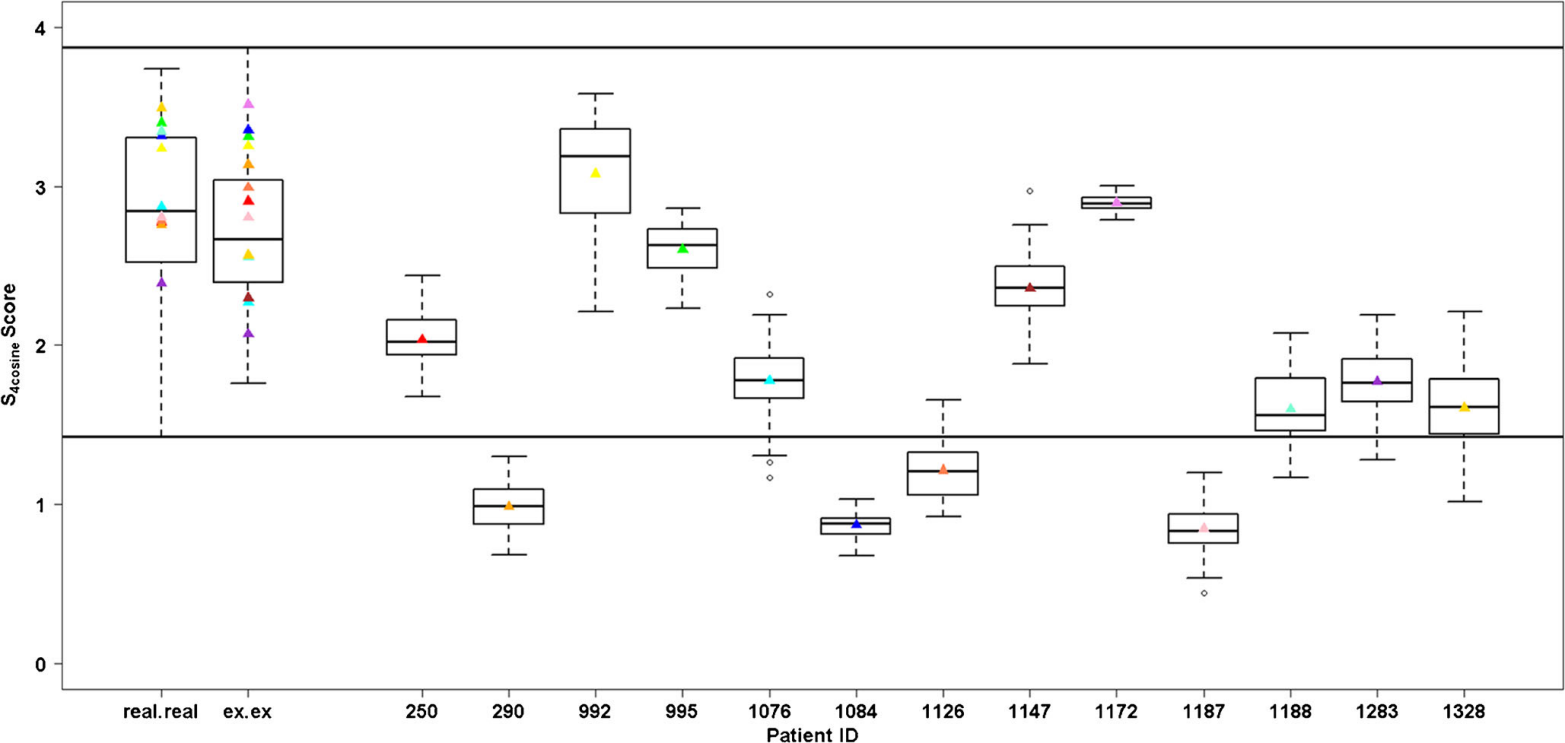
Box plots of the S_4cosine_ score (fit, retrofit, cosine, overlap). The first two box plots represent the intra-patient comparison of the spectra from all the *real* (first box plot) and ex vivo (second box plot) ROIs. All the other box plots represent the inter-sample comparisons for each patient. The box contains data that fall between the first and third quartiles, the horizontal line indicates the median, and the brackets delineate 1.5 times the interquartile range (with data outside this range defining outliers). Coloured triangles represent the average score value of all the comparisons in each patient

To quantify the degree of similarity, the S_4cosine_ score system was calculated for all the possible paired comparisons for each patient. Firstly, S_4cosine_ was calculated for both intra-sample and inter-sample ROI comparisons for all patients and the results are summarized in ESM Tables S1–S2. The intra-sample comparisons of the real and ex vivo FNA are reported in the first two box plots of Fig. 4 and were calculated in order to construct our reference interval of equivalence, which is represented as horizontal lines in the figure.

When the distribution of inter-sample comparisons for each patient was compared to the range of equivalence of the intra-sample intervals, no remarkable differences were observed for patients P250 (mean S_4cosine_ = 2.03), P992 (mean S_4cosine_ = 3.08), P995 (mean S_4cosine_ = 2.60), P1147 (mean S_4cosine_ = 2.36), and P1172 (mean S_4cosine_ = 2.90); in fact, the inter-sample comparison ranges were completely comprised in the intra-sample ranges (S_4cosine_ min-max = 1.43–3.87). However, heavy dissimilarities for patients P290 (mean S_4cosine_ = 0.98), P1084 (mean S_4cosine_ = 0.87), P1126 (mean S_4cosine_ = 1.21), and P1187 (mean S_4cosine_ = 0.85) were observed, while at least a 75% of overlap was found for patients P1076 (mean S_4cosine_ = 1.78), P1188 (mean S_4cosine_ = 1.60), P1283 (mean S_4cosine_ = 1.77), and P1328 (mean S4cosine = 1.61) resulting in being right over the lower limit of the reference range.

The similarity score value mirrored the qualitative evaluation based on mass spectra profiles (Fig. 1). In Fig. 1a, the mass spectra profiles of patients with the highest similarity score are shown, whereas in Fig. 1c, those with the lowest similarity are presented. The overlap index of the *real* and ex vivo average mass spectra of the ROIs are depicted in Fig. 1 and the calculated values, for all the 13 comparisons, ranged between 32 and 78% (ESM Table S1). The 4 patients in Fig. 1c (P290, P1084, P1126, and P1187), with the lowest similarity score, were also those with the lowest overlap, having a mean value of 37% ± 5%. However, for 9 out of the 13 patients compared, the profile of real and ex vivo spectra were better conserved (Fig. 1a and b), where the mean overlap value was 65% ± 8%.

## Discussion

*Real* and ex vivo FNA samples have been largely used in recent years to investigate several pathologies such as thyroid, breast, lung, and cervical cancers [3, 8–11]. The high value of *real* biopsies is related to the fact that it is the first-line diagnostic procedure, as in the diagnosis of thyroid nodules [12–14]. On the other hand, ex vivo samples are easily available, since their collection does not directly involve the patients, but only the surgical specimens, with the consequence that the amount of ex vivo samples is not influenced by collection-driven variability from patient to patient.

In recent years, there has been growing interest in the evaluation of the molecular similarity of *real* and ex vivo FNA specimens. Wong et al. performed a microarray data analysis, comparing pre- and post-surgery breast FNAs from the same patients showing that the FOS-related genes were differentially expressed before and after surgery and underlining a different gene expression profiles depending on the timing of sample collection [15].

On the contrary, Ciregia et al. suggested a possible similarity of the proteome of pre- and post-surgery thyroid FNAs using pooled samples from different benign and malignant nodules investigated by bidimensional electrophoresis (2DE) maps [7]. Furthermore, they evaluated, by 2DE, Western blot analysis, and ELISA, the levels of five proteins previously found to be upregulated in malignant post-surgery FNAs [16], demonstrating similar results for ANXA1 and LDHB, whereas a not clear similarity was shown for Moesin, FLC, and FHC [7]. This apparent discrepancy of the levels of the 5 proteins could derive from the biological variability between patients. Notwithstanding, the similarity of the intra-patient proteomic profiles of these FNA thyroid samples was not proven.

Among the several proteomics approaches available to investigate the tissue proteome in order to enlighten specific proteomic profiles able to distinguish different specimens, the MALDI-MSI appears to be successful even if less informative than others (e.g. bottom-up approach). In particular, MALDI-MSI technique provides the possibility to (i) analyse cytological samples without compromising sample morphology and (ii) use for the statistical analysis only areas containing the cells of interest without losing information due to the complete protein extraction.

Indeed, in the last years, the proteomic profiles of cytological thyroid samples have also been investigated using a MALDI-MSI approach which allows the localization of proteins to be preserved inside the samples, without the need for full extraction of the proteome [4, 17, 18]. It has been shown that *real* FNAs could present a higher amount of red blood cells, mostly due to the sample collection procedure and the cutaneous vasculature of the neck that cause a high percentage of inadequate samples for cytological diagnosis [5]. The presence of haemoglobin represents an issue during MALDI-MSI proteomic analysis, interfering with the ionization process. In fact, in the worst cases, the high amount of haemoglobin in the specimens suppressed any other protein signals. This issue was overcome using a liquid-based sample preparation procedure, allowing both ex vivo and *real* specimens to be successfully used for MALDI-MSI analysis [5, 6].

In 2019, DeHoog et al. observed that the FNA collection method did not influence the DESI-MSI lipid profiles when comparing samples collected from the same nodule (preoperative and post-surgery) [3]. They had FNAs collected during both routine outpatient biopsy and post-surgery for only two patients, and, for one patient, their classification model did not provide the same prediction from the two collection methods. This discrepancy was explained by the different number of pixels extracted from the samples and the different amount of thyrocyte cell clusters [3]. Similarly, in the current study, we observed that when *real* and ex vivo samples differed in terms of the amount of thyrocyte clusters (Fig. 3c, d), the similarity of the proteomic profiles was negatively affected (Fig. 1c). On the other hand, when the morphology was comparable (Fig. 3a, b), the proteomic profiles were highly similar from both a qualitative (Fig. 1a) and quantitative standpoint (Fig. 4).

We have preliminarily shown in a pilot study that the mass spectra of thyrocyte cell clusters of *real* FNAs are useful in correctly predicting the nature of the thyroid lesions [2]. However, we have also noticed that the classification of the proteomic profiles of both ROIs and *pixel-by-pixel* from *real* samples was influenced by the paucity of thyrocyte cell clusters. Indeed, patients P1084, P1126, and P1187, who present a *real* vs. ex vivo S_4cosine_ mean of 0.87, 1.21, and 0.85, respectively, were misclassified as benign by our classification model [2]. Moreover, the model was able to correctly classify the samples as malignant when using the ex vivo FNA from the same patients, showing that the discriminant features found by the model, built using *real* FNAs, were still expressed in the ex vivo counterparts.

To our knowledge, this is the first study that evaluates the mass spectra similarity of intra-patient proteomic profiles of *real* thyroid needle washes and ex vivo thyroid FNAs. Spectra similarity was evaluated using a composite index that integrates different aspects of the proteomic profiles: the degree of common peaks (fit and retrofit), the association between intensities (cosine) and the whole shape (overlap). In our study, the main contribution to these indices mainly derived from the overlap and the cosine components, as shown in ESM Fig. S3 and Table S1.

The limited number of patients included in this study could be considered a limitation. However, in this study, we decided to investigate both *real* and ex vivo specimens from the same patient. These represent the specimens providing the direct comparison, instead of using two different groups of patients (one for *real* and one for the ex vivo), but both samples are not always available. In fact, it should be considered that it is not uncommon that patients refer to different healthcare facilities during their follow-up. Despite that, the intra-patient comparison still clearly enlightens that the sample collection method of FNAs (*real* needle washes and ex vivo) does not influence the proteomic profile itself, but the discrepancies observed between samples were solely related to the amount of cellular content. The possibility to use data from different FNA sample types will avoid losing cases whenever *real* samples are not available (e.g. due to reasons related to technical or sample quality), thus allowing the data obtained from both samples to be integrated to increase the sample size and hence construct powerful statistical models.

## Supplementary information

ESM 1 (PDF 386 kb)

ESM 2 (XLSX 37 kb)

ESM 3 (XLSX 14 kb)
