# Supplementary material for: Ex vivo thyroid fine needle aspirations as an alternative for MALDI-MSI proteomic investigation: intra-patient comparison
Source: Anal Bioanal Chem. 2020 Dec 5;413(5):1259–66. doi: 10.1007/s00216-020-03088-4 (PMC7892726; doi:10.1007/s00216-020-03088-4)
Supplement: Supplementary file 1 — (PDF 386 kb) [file 216_2020_3088_MOESM1_ESM.pdf]

Supplementary Information

Ex vivo thyroid fine needle aspirations as an alternative for MALDI-MSI proteomic investigation: intra-patient comparison

Isabella Piga[1#*], Giulia Capitoli[2#], Francesca Clerici[1], Allia Mahajneh[1], Virginia Brambilla[3], Andrew Smith[1], Davide Leni[4], Vincenzo L'Imperio[3], Stefania Galimberti[2], Fabio Pagni MD[3], Fulvio Magni[1]

[1] Proteomics and Metabolomics Unit, School of Medicine and Surgery, University of Milano - Bicocca, Vedano al Lambro, Italy

[2] Bicocca Bioinformatics Biostatistics and Bioimaging B4 Center, School of Medicine and Surgery, University of Milano - Bicocca, Monza, Italy;

[3] Pathology, School of Medicine and Surgery, University of Milano - Bicocca, San Gerardo Hospital, ASST Monza, Italy

[4] Radiology, San Gerardo Hospital, ASST Monza, Italy

# First equally contributed authors

*Corresponding author:

Dr. Isabella Piga, PhD

email: isabella.piga@unimib.it

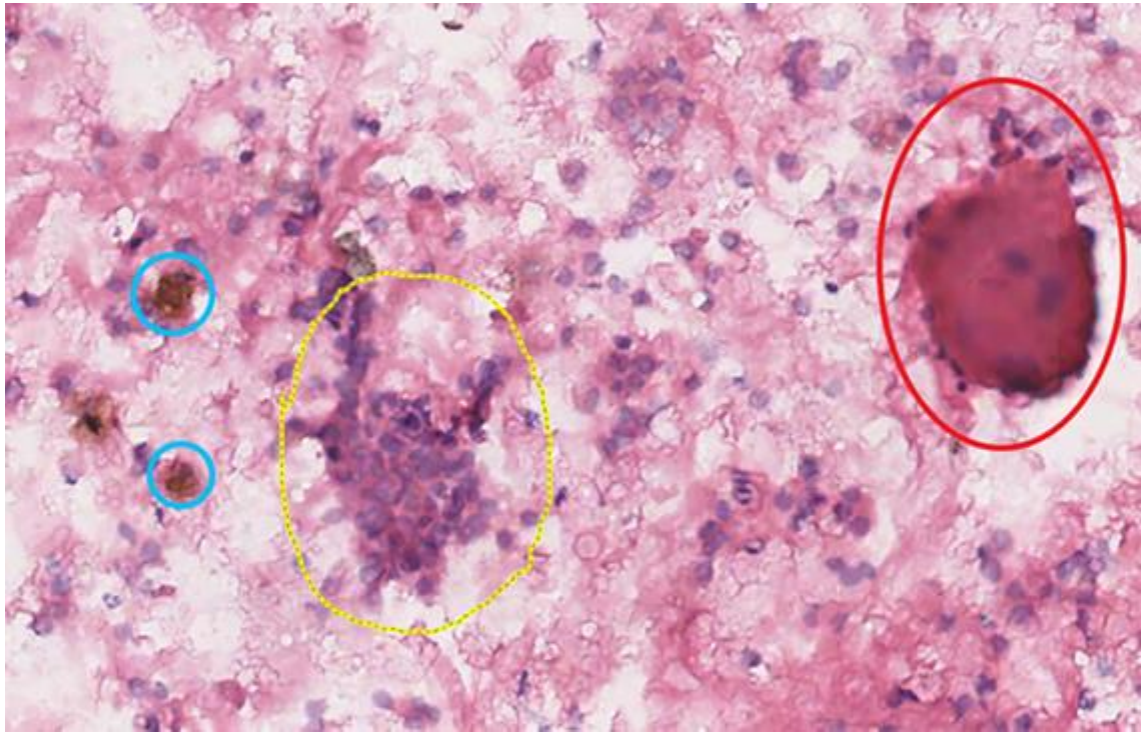

Fig. S1 For each cytological specimen the pathologist annotated the ROIs for proteomic analysis selecting those areas characterized by aggregates/clusters purely composed by thyrocytes (yellow circle) without additional interfering elements that could affect the proteomic analysis, such as colloid material (red circle) and inflammatory cells (e.g. hemosiderin laden macrophages, blue circles).

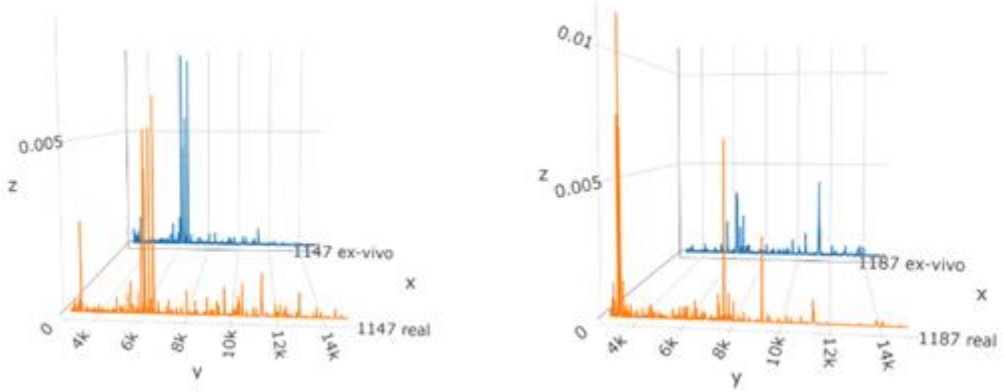

Fig. S2 a 1-by-1 figure of the comparison between the average mass spectra obtained from *real* and *ex-vivo* ROIs from the same patient of the most similar (P1147) and the most dissimilar (P1187) spectra.

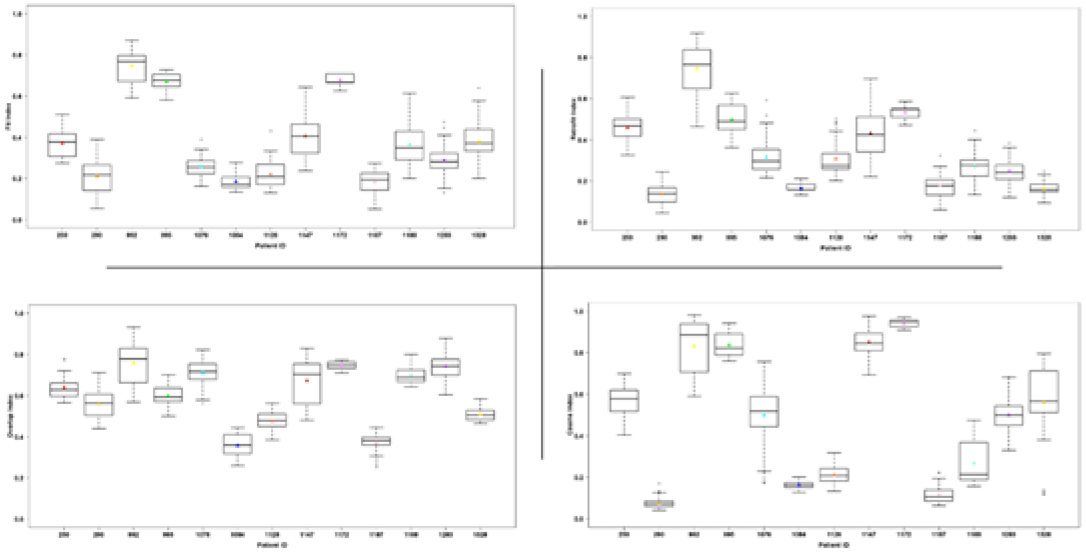

Fig. S3 Box-plots of the fit, retrofit, overlap, and cosine $S_{4cosine}$ components.

Table S1 and Table S2 see separate Excel files
